# CBCT-based Online Adaptive Radiotherapy for Prostate Cancer: Dosimetrical Aspects and Comparison to Non-Adaptive Conventional IGRT

**DOI:** 10.1177/15330338251405772

**Published:** 2026-01-12

**Authors:** Niklas Christian Scheele, Jann Fischer, Lovis Hampe, Tim Niemeier, Jessica Moldauer, Daniela Schmitt, Manuel Guhlich, Martin Leu, Leif Hendrik Dröge, Arne Strauß, Stefan Rieken, Laura Anna Fischer, Rami Ateyah El Shafie

**Affiliations:** 1 Department of Radiotherapy and Radiation Oncology, 74922 University Medical Center, Göttingen, Germany; 2 Comprehensive Cancer Center, University Medical Center, Göttingen, Germany; 3 Department of Urology, University Medical Center, Göttingen, Germany

**Keywords:** online adaptive radiotherapy (oART), image guided radiotherapy (IGRT), intensity modulated radiotherapy (IMRT), prostate cancer, target coverage, organs at risk (OARs)

## Abstract

**Introduction:**

Daily anatomical variations in prostate cancer radiotherapy, particularly due to pelvic organ motion and filling, can compromise target coverage and increase exposure to organs at risk (OARs). Conventional image-guided radiotherapy (IGRT) uses fixed safety margins and daily couch corrections to account for these variations, potentially leading to overtreatment of healthy tissue or insufficient tumor coverage. Online adaptive radiotherapy (oART), based on cone-beam computed tomography (CBCT), enables daily plan adaptation to the patient's anatomy, offering improved precision, enhanced target coverage, and better OAR sparing. This retrospective study compares oART to conventional IGRT in prostate cancer treatment.

**Methods:**

A total of 153 treatment fractions from six consecutive prostate cancer patients treated with oART on a Varian Ethos system were analyzed. For each fraction, three plans were evaluated: the scheduled plan (initial plan recalculated on daily CBCT), the adapted plan (reoptimized based on daily anatomy), and the verification plan (applied dose recalculated on a post-adaptation CBCT). Dose–volume metrics for target volumes and OARs were assessed, and clinical acceptability was evaluated. Interfractional prostate volume changes and treatment times were examined.

**Results:**

CTV D_98%_ improved significantly with adaptation (median 97.85% to 98.55%; p < 0.01) and further increased in the verification plan (98.8%; p < 0.01), alongside reduced interquartile ranges. PTV D_98%_ rose from 90.1% to 97.1% with adaptation and to 96.9% after verification (p < 0.01). Bowel and bladder doses showed dosimetrical advantage. Clinically acceptable plans increased from 24.8% (scheduled) to 98% (adapted) and 85.6% (verification). Scheduled plans were not used clinically. Median prostate volume remained stable despite inter-individual variation. oART required about twice the treatment time of IGRT.

**Conclusion:**

Although more time-consuming, oART improved target dose coverage and optimized OAR sparing, while simultaneously reducing dose variability for both the target and some OARs compared to IGRT. The plan acceptability improved significantly.

## Introduction

In conventional image-guided radiotherapy (IGRT), a computed tomography (CT) scan of the tumor region is acquired prior to treatment initiation. This imaging data serves to delineate the target volume as well as adjacent organs at risk (OARs). Based on these images, a patient-specific radiotherapy plan is generated. Once established, the treatment plan is applied consistently throughout the course of therapy.

To correct for patient positioning errors, three-dimensional cone-beam CT (CBCT), integrated into the treatment system, is used to adjust translational and possibly rotational misalignments by modifying the treatment couch position.^1,2^ However, conventional IGRT does not account for daily anatomical variations in the target region, which are typically managed by introducing large safety margins around the clinical target volume (CTV).^3^ This approach, while ensuring adequate tumor coverage, inevitably increases radiation exposure to surrounding healthy tissues, possibly raising the risk of therapy-induced toxicities.^4,5^

This challenge is particularly pronounced in pelvic tumors, where substantial safety margins are required to compensate for variations in bladder and rectal filling.^6–8^ To overcome these limitations, online Adaptive Radiotherapy (oART) has emerged as an advanced technique that enables real-time adaptation of treatment plans. In this approach, a new radiotherapy plan is generated daily based on CBCT imaging acquired immediately before each treatment session. The integration of artificial intelligence (AI) facilitates automated segmentation, dose calculation, and real-time plan optimization, allowing for precise adaptation to daily anatomical and morphological changes while also correcting for translational and rotational errors.^9^ By dynamically adjusting the treatment plan to the patient's current anatomy, oART can prospectively reduce safety margins, thereby potentially lowering the risk of therapy-related toxicities.^10^

Despite these advantages, clinical evidence remains limited regarding which patient populations and tumor types derive the greatest benefit from oART and its effectiveness in prostate cancer treatment is still under investigation.^11,12^ This study aims to evaluate the potential benefits of oART in prostate cancer by comparing its dose distribution with that of conventional IGRT.

## Materials, Patients and Methods

### Study Design and Patient Selection

This retrospective analysis presents data following the implementation of oART using the Ethos system (Varian Medical Systems, Palo Alto, CA, USA) at a single tertiary cancer center in Germany, initiated in January 2023. The employed version (1.1) of the Ethos system enables AI-supported kV cone-beam CT-based radiotherapy using a linear accelerator. In total, 155 treatment fractions were applied to six patients, but due to technical reasons, data from two fractions could not be evaluated, as retrospective data extraction was defective. Consequently, the final analysis was based on 153 fractions applied to the prostate (CTV) with an isotropic 5 mm margin (PTV). Five patients received a total radiation dose of 67.5 Gy, administered in 27 fractions, while one patient underwent a total dose of 55 Gy**,** delivered in 20 fractions**.** All patients were retrospectively included based on treatment slot availability following the clinical implementation of the workflow. The study was approved by the local ethics committee (approval number 7/8/23) and conducted in accordance with the Declaration of Helsinki. All patient data were fully anonymized prior to analysis to ensure confidentiality and prevent individual identification.

### Workflow Description

Data acquisition for this analysis was done following a standardized clinical workflow for oART, published previously.^5^ In summary, it commenced with the acquisition of the planning CT (pCT) scan, serving as the foundation for subsequent steps. An AI-driven auto-segmentation tool (Limbus Contour, v 1.8.0-B3, Limbus AI Inc., Regina, SK, Canada) was employed to delineate organs at risk (OARs), ensuring initial contouring automation. If necessary, manual refinements were applied by radiation oncologists, who also performed target volume delineation.

Following this, the treatment intent was established within the Ethos treatment planning software (v 2.1), incorporating key parameters such as fractionation schedule, prescribed dose, and adaptation criteria, alongside predefined dose-volume constraints for OARs. Multiple treatment plans were then generated using intensity-modulated radiation therapy (IMRT) techniques, which were systematically evaluated. If none met the required standards, plan regeneration (with adjusted parameter) and plan selection were iteratively conducted until a satisfactory plan was identified and approved.

Each treatment session commenced with the acquisition of a CBCT scan to facilitate IGRT. For standard IGRT, couch positional adjustments were made based on the CBCT before irradiation. In the context of oART, the pCT was deformably registered to the CBCT, yielding a synthetic CT (sCT). This sCT preserved the Hounsfield unit values of the pCT while conforming to the anatomical geometry of the CBCT, thereby ensuring accurate dose calculations within the adaptive workflow.

The next phase involved AI-assisted recontouring of OARs, with optional manual corrections to ensure accuracy. Verified OAR contours enabled the automatic transfer of original target volumes onto the CBCT via deformable registration, with radiation oncologists retaining the ability to adjust contours as needed. Subsequent recalculation of dose distributions yielded two competing treatment plans: (1) the scheduled plan (SCH), which represented the recalculated original plan on the sCT with initial automatic couch shift adjustments, and (2) the adapted plan (ADP), a newly optimized treatment plan that accounted for anatomical variations observed in the daily CBCT.

A physician then reviewed both plans and selected the most appropriate option, which was subsequently approved after an independent dose verification by a medical physicist. As an additional quality assurance step, an optional verification CBCT (VER) was performed post-approval to assess potential peri-processual patient movement, allowing for further conventional IGRT-based couch corrections if required.

Finally, the patient was treated according to the selected plan, either SCH or ADP. Post-treatment, the delivered dose was reconstructed using the verification CBCT (VER) to ensure dosimetric accuracy and treatment fidelity.

### Plan Generation, Endpoints and Statistics

For all patients, treatment planning was conducted following the local clinical protocol. This included a planning CT (pCT) scan performed with a Brilliance BigBore CT (Philips Healthcare), utilizing a slice thickness of 3 mm. Patients were advised to maintain a comfortably filled bladder and an emptied rectum, both for the pCT and during the radiotherapy sessions. To facilitate adherence to these requirements, they were provided with written instructions regarding appropriate dietary, hydration and voiding practices. The dose-volume specifications applied in radiotherapy planning for both target volumes and OARs are detailed in Table 1. For the rectum D_0.1cc_ parameter, the relative dose – normalized to the prescription dose of the target – was evaluated to ensure comparability across different dose regimens.

**Table 1.** Dose-Volume-Histogram (DVH) Parameter for Organs at Risk and Target Volumes. ALARA: As Low as Reasonably Achievable.

Organ	Dose-Volume-Specification	Clinical Standard
CTV	D_98%_	≥95%
PTV	D_98%_	≥95%
PTV	D_2%_	≤105%
Rectum	D_mean_	ALARA
Rectum	D_0.1cc_	<66 Gy
Rectum	V_40Gy_	ALARA
Bowel	D_0.1cc_	<66 Gy for 67.5 Gy (27 fx) pat. <55 Gy for 55 Gy (20 fx) pat.
Bladder	D_mean_	ALARA
Bladder	V_40Gy_	ALARA

Anatomical variations among patients occasionally led to partial overlap between intestinal loops and the PTV. To comply with the bowel dose constraint D_0.1cc_, as outlined in Table 1, the PTV were adjusted according to the following principle:

PTV=(CTV+5mm)–Bowel

Whenever bowel loops were adjacent to the PTV, the overlapping area was excluded while still maintaining full CTV coverage.

The patients underwent intensity-modulated radiotherapy (IMRT) using either 9 or 12 equally spaced fields. Volumetric modulated arc therapy (VMAT) was not implemented in routine clinical practice due to the extensive time required for both calculation and optimization. The initial treatment plan generation followed the same process for both oART and IGRT.

To assess prostate volumes, CTV data were collected and normalized to the initial treatment plan, ensuring comparability while accounting for interfractional variations. Regarding acceptability, each treatment fraction was assessed according to clinical standards for CTV and PTV, with a D_98%_ value above 95% of the prescribed dose regarded as acceptable.

Treatment duration was analyzed for both techniques. For oART, the time from acquisition of the first CBCT to the verification CBCT was measured for each fraction. For IGRT, the corresponding interval from the initial CBCT to beam delivery was recorded in a separate cohort treated during the same period.

### Statistical Analysis

The comparison of treatment times between oART and IGRT was performed using the Mann–Whitney U test (two-sided). The null hypothesis was that time needs were not divergent, with p-values below 0.05 regarded as statistically significant. Fraction acceptability was analyzed using the McNemar test, assuming the null hypothesis that no difference existed in acceptability, with statistical significance defined at p < 0.05. Dose and volume parameters for target structures and organs at risk were statistically analyzed using the Wilcoxon signed-rank test, employing a paired design with patients serving as their own controls. The null hypothesis stated that no significant differences existed between the evaluated treatment plans. A two-sided significance threshold of p < 0.05 was applied. The statistical computations were performed in Python (v3.12) utilizing the packages pandas (v2.2.1), pydicom (v2.4.4), and scipy (v1.12).

All relevant methodological details have been provided to ensure reproducibility by other researchers. The reporting of this study conforms to the STROBE guidelines.^13^

## Results

### Patient Selection

As part of this analysis, six patients undergoing prostate radiotherapy were included. The median age was 71.5 [first quartile (Q1): 71.3; third quartile (Q3): 80.25]. The time period ranged from October 2023 to March 2024, during which 155 fractions were delivered using IMRT. Due to technical reasons, data from two fractions were excluded because retrospective extraction was defective. While five patients each received a total dose of 67.5 Gy (27 fractions), one patient received 55 Gy (20 fractions). In all cases, the adaptive plan was selected instead of the scheduled plan.

#### Target Volumes–Volume Differences

**Figure 1.**
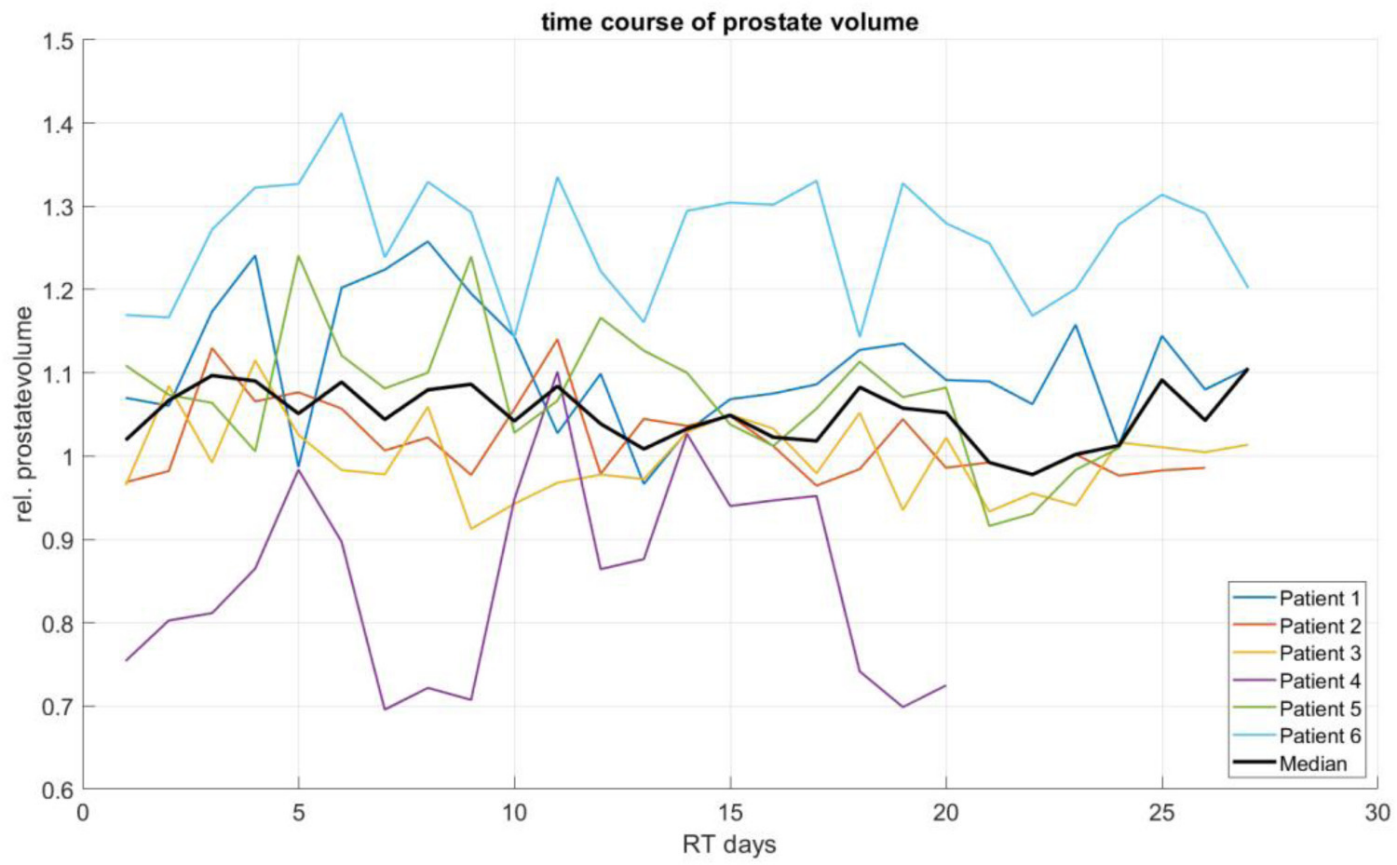
Temporal evolution of prostate volume across all six patients during radiotherapy. Individual patient curves are shown alongside the median volume (black line).

The analysis of prostate volume changes over the course of radiotherapy revealed substantial inter-patient variability. As shown in Figure 1, some patients exhibited an increase in prostate volume throughout treatment, while others showed a decreasing trend or considerable fluctuations between fractions. Despite these distinct individual trends, the median prostate volume remained relatively stable over time, without indicating a clear upward or downward trajectory. Throughout the treatment period, the median fluctuated within a narrow range, with an overall median of 1.05 (Q1: 0.98; Q3: 1.14).

### Target Dose Differences

**Figure 2.**
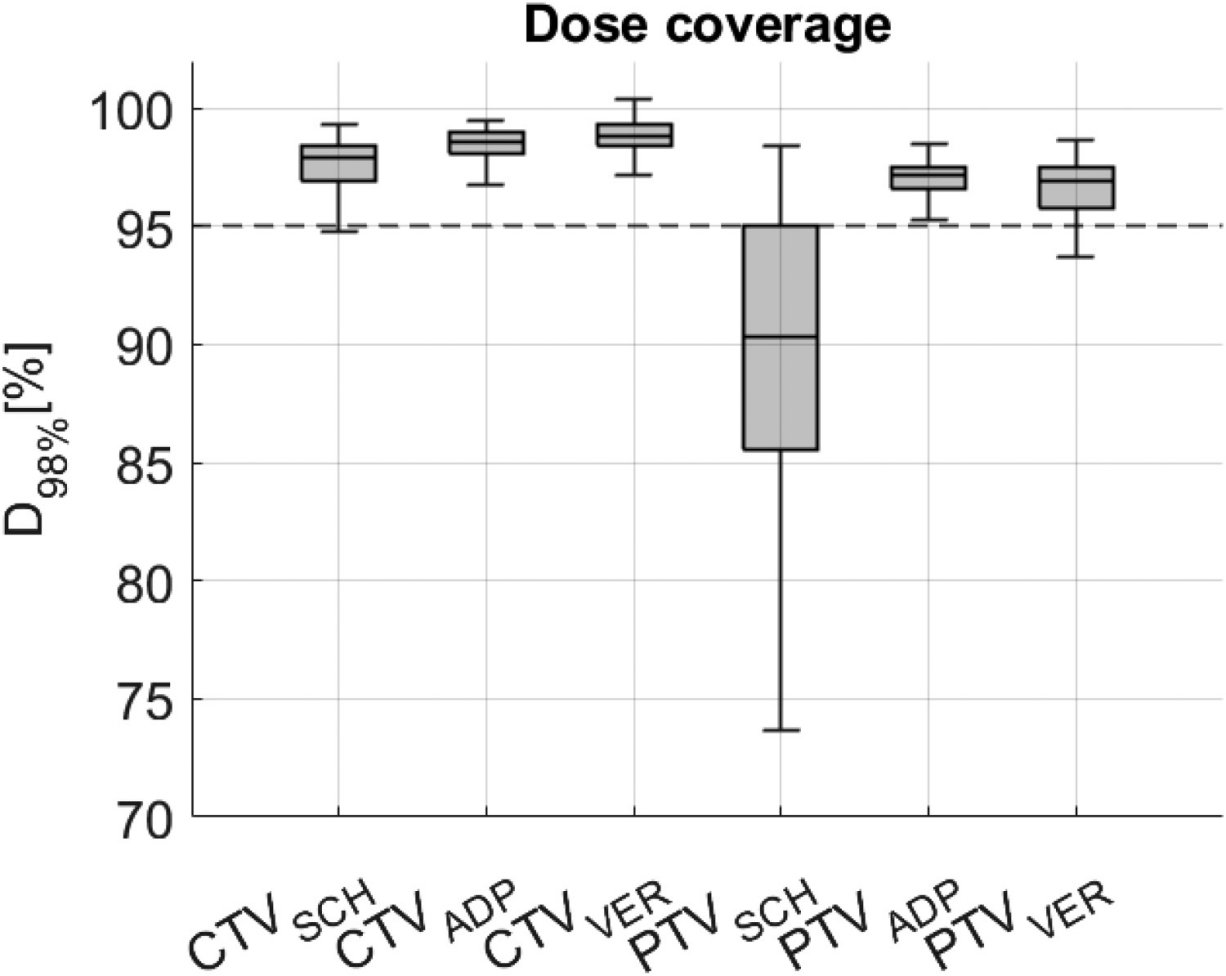
The D_98%_ dose coverage for scheduled (SCH), adapted (ADP), and verification (VER) doses is displayed for the CTV and PTV, encompassing all patients. The internal clinical standard threshold (D_98%_ > 95%) is represented by an interrupted line.

The analysis of the CTV dose coverage using the D_98%_ parameter revealed a median value of 97.9% for SCH, 98.6% for ADP, and 98.8% for VER. Regarding the PTV, the median D_98%_ was 90.1% for SCH, 97.1% for ADP, and 96.9% for VER. As shown in Table 2 and graphically demonstrated in Figure 2, the comparisons between SCH and ADP as well as SCH and VER revealed highly significant differences for both CTV (p < 0.01) and PTV (p < 0.01). Moreover, the IQR decreased when comparing SCH to ADP and SCH to VER. With respect to the CTV, the D_98%_ IQR decreased from 1.7 for SCH to 0.9 for both ADP and VER. For the PTV, the IQR decreased from 9.17 for SCH to 0.9 for ADP and 1.75 for VER.

**Table 2.** Statistical Data on CTV and PTV (D_98%_ and D_2%_) for SCH, ADP, and VER Plans, Including Median Values, First Quartile (Q1), Third Quartile (Q3), Interquartile Range (IQR, Q3-Q1), and p-Values from the Wilcoxon Signed-Rank Test.

	Parameter	Plan	Median [%]	Q1 [%]	Q3 [%]	IQR	p-value (to SCH)	p-Value (to ADP)
CTV	D_98%_	SCH	97.9	96.7	98.4	1.7
		ADP	98.6	98.1	99	0.9	< 0.01
		VER	98.8	98.4	99.3	0.9	< 0.01	< 0.01
PTV	D_98%_	SCH	90.1	85.53	94.7	9.17
		ADP	97.1	96.6	97.5	0.9	< 0.01
		VER	96.9	95.73	97.48	1.75	< 0.01	< 0.01
	D_2%_	SCH	102.5	101.9	102.9	1
		ADP	102.9	102.5	103.3	0.8	< 0.01
		VER	103.2	102.9	103.6	0.7	< 0.01	< 0.01

**Figure 3.**
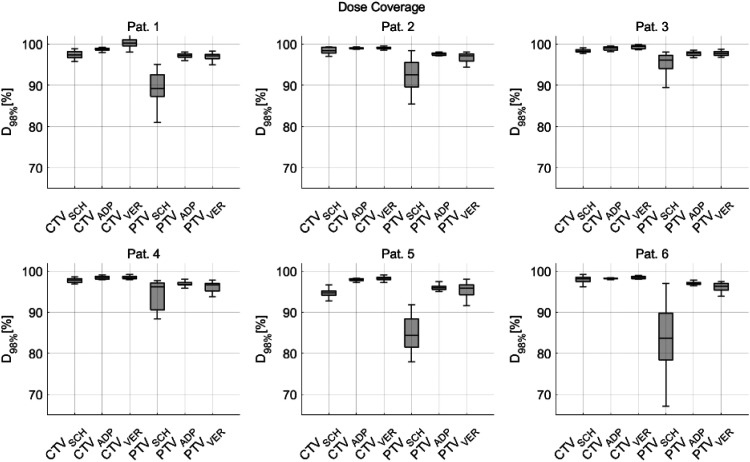
The dose coverage (D_98%_) of CTV and PTV for scheduled (SCH), adapted (ADP), and verification (VER) dose for all six individual patients.

Figure 3 demonstrates the presence of individual differences when analyzing isolated patients.

**Figure 4.**
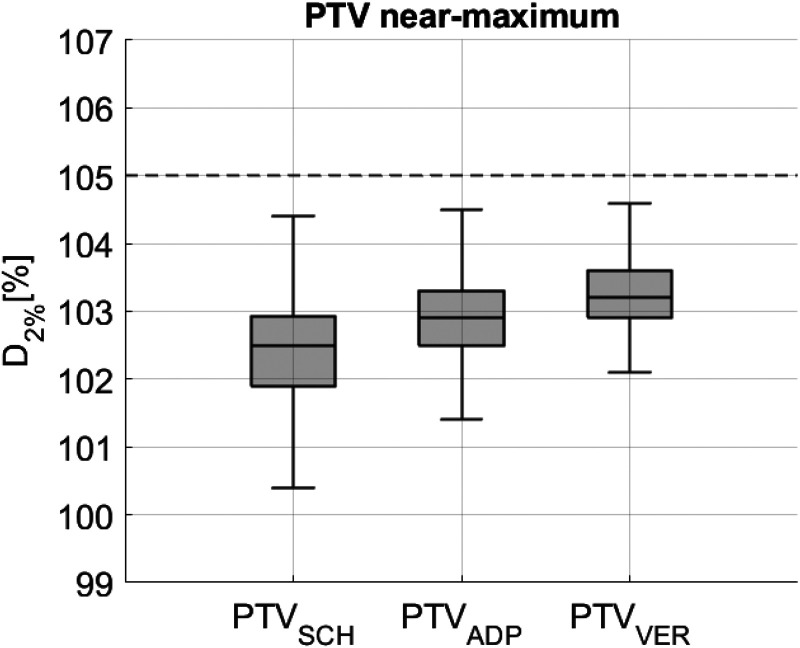
The PTV near-maximum (D2%) parameter for scheduled (SCH), adapted (ADP) and verification dose combined for all patients. The internal clinical standard constraint (D2% ≤ 105%) is represented by the interrupted line.

Regarding the PTV near-maximum dose, represented by D_2%_, the median was 102.5% for SCH, 102.9% for ADP, and 103.2% for VER. The differences between SCH and ADP, SCH and VER, as well as ADP and VER were all found to be significant, with p < 0.01 in each case. These differences are depicted in Figure 4. The dashed black line represents the acceptable cutoff of 105%, according to the internal clinical standard. Additionally, a consistent decrease in the IQR from SCH (1.0) to ADP (0.8) and VER (0.7) can be observed.

A detailed statistical analysis of target doses can be found in Table 2.

Given that the clinically acceptable range is defined by D_98%_ of the CTV and PTV exceeding 95% of the prescribed dose, while D_2%_ of the PTV remains below 105%, the proportion of fractions satisfying these criteria differed substantially among the evaluated planning approaches. Specifically, 24.8% (38 out of 153) of fractions from SCH plans, 98% (150 out of 153) from ADP plans and 85.6% (131 out of 153) from VER plans met these dosimetric constraints. This demonstrates statistically significant enhancement in plan acceptability when transitioning from SCH to ADP as well as from SCH to VER, as confirmed by the McNemar test (p < 0.01 for both comparisons). Nevertheless, a significant decline in acceptability was observed when shifting from ADP to VER (p < 0.01). Despite this acceptability criterion, the ADP plans were utilized in clinical practice.

**Figure 5.**
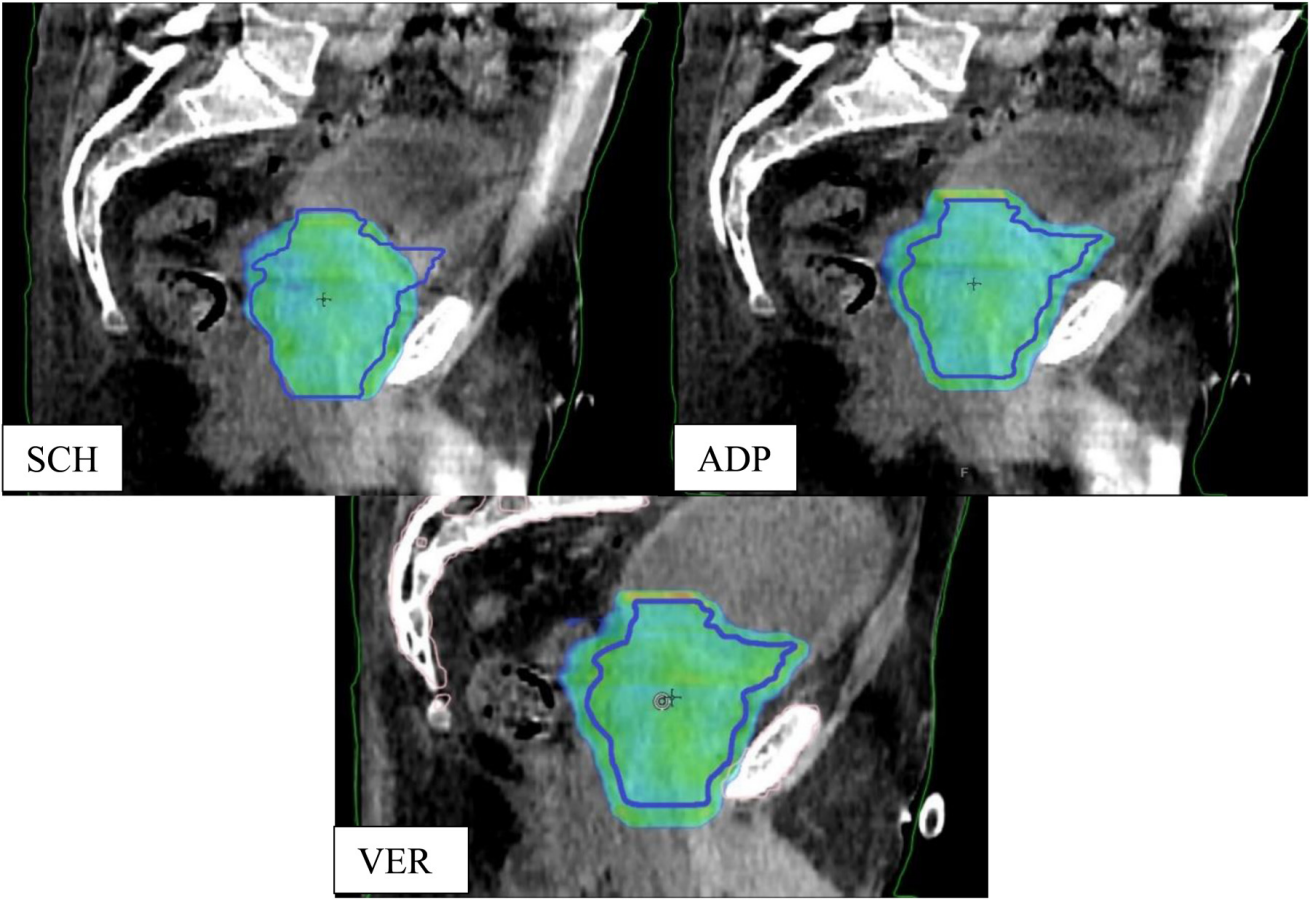
Shows the visualized 95% isodose for the scheduled plan (SCH, top left), the adapted plan (ADP, top right) and the reconstructed verification plan (VER, bottom) of pat. 6, fraction 11. The blue line represents the CTV.

Figure 5 illustrates the dose distribution across the three planning approaches (SCH, ADP and VER) highlighting differences in target coverage and the impact of anatomical variations. In the SCH plan, portions of the CTV (outlined in blue) received doses below the prescribed level, indicating suboptimal target coverage. Following anatomical modifications, the ADP plan demonstrated improved dose distribution, effectively enhancing covering of the CTV. In contrast, the VER plan exhibited minor deviations from the ADP plan, attributable to intrafractional anatomical changes.

### Organs at Risk–Dose Differences

**Figure 6.**
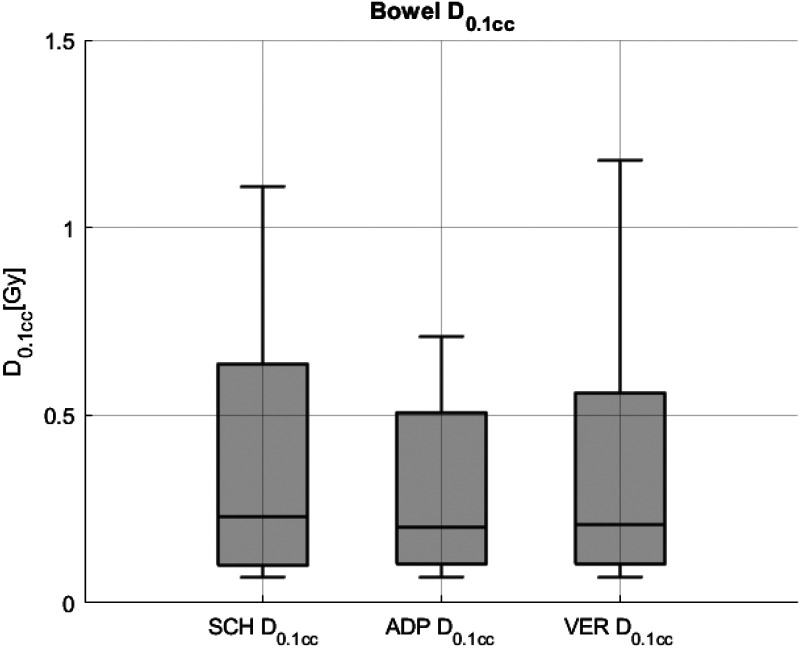
Bowel dose exposure measured as D0.1cc for scheduled (SCH), adapted (ADP), and verification (VER)plans.

Statistically significant differences in bowel D_0.1cc_ were observed between SCH and ADP, SCH and VER (p < 0.01 for both), as well as between ADP and VER (p = 0.02). The median D_0.1cc_ was 0.23 Gy/fx for SCH, compared to 0.2 Gy/fx for ADP and 0.23 Gy/fx for VER. The IQR for bowel D_0.1cc_ decreased from 0.55 for SCH to 0.41 for ADP and 0.48 for VER. These findings are illustrated in Figure 6, which depicts the bowel D_0.1cc_ for SCH, ADP, and VER plans.

Respective statistical data are detailed in Table 3.

**Table 3.** Statistical Data for the Bowel (D0.1 cc) for SCH, ADP, and VER Plans. Median, First Quartile (Q1), Third Quartile (Q3) Interquartile Range IQR (Q3-Q1) and p-Values (Wilcoxon Signed-Rank Test) are Included.

	Parameter	Plan	Median [Gy/fx]	Q1 [Gy/fx]	Q3 [Gy/fx]	IQR	p-Value (to SCH)	p-Value (to ADP)
Bowel	D_0.1cc_	SCH	0.23	0.10	0.65	0.55
		ADP	0.20	0.11	0.52	0.41	<0.01
		VER	0.23	0.11	0.59	0.48	<0.01	0.02

**Figure 7.**
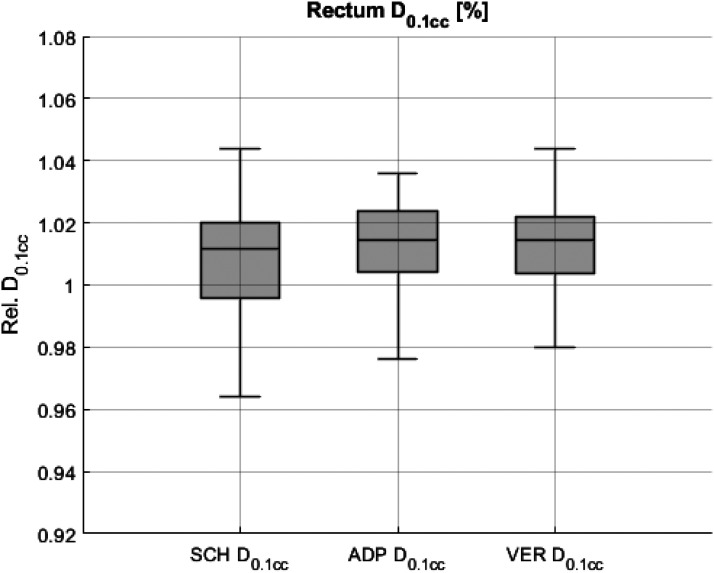
Rectum dose exposure measured as relative D_0.1cc_ for scheduled (SCH), adapted (ADP), and verification (VER) plans.

For rectum relative D_0.1cc_ no significant differences were found between SCH, ADP and VER. The median relative D_0.1cc_ was 1.011 for SCH and 1.015 for ADP and VER. As shown in Figure 7, the IQR for rectum relative D_0.1cc_ decreased from 0.024 for SCH to 0.020 for ADP and increased to 0.060 for VER.

Respective statistical data are detailed in Table 4.

**Table 4.** The Table Displays Statistical Data for the rectum (D0.1 cc) for SCH, ADP, and VER Plans. Median, First Quartile (Q1), Third Quartile (Q3) Interquartile Range IQR (Q3-Q1) and p-Values (Wilcoxon Signed-Rank Test) are Included.

	Parameter	Plan	Median [Gy/fx]	Q1 [Gy/fx]	Q3 [Gy/fx]	IQR	p-Value (to SCH)	p-Value (to ADP)
Rectum	D_0.1cc_ [%]	SCH	1.011	0.996	1.020	0.024
		ADP	1.015	1.004	1.024	0.020	0.17
		VER	1.015	1.004	1.064	0.060	0.06	0.07

**Figure 8.**
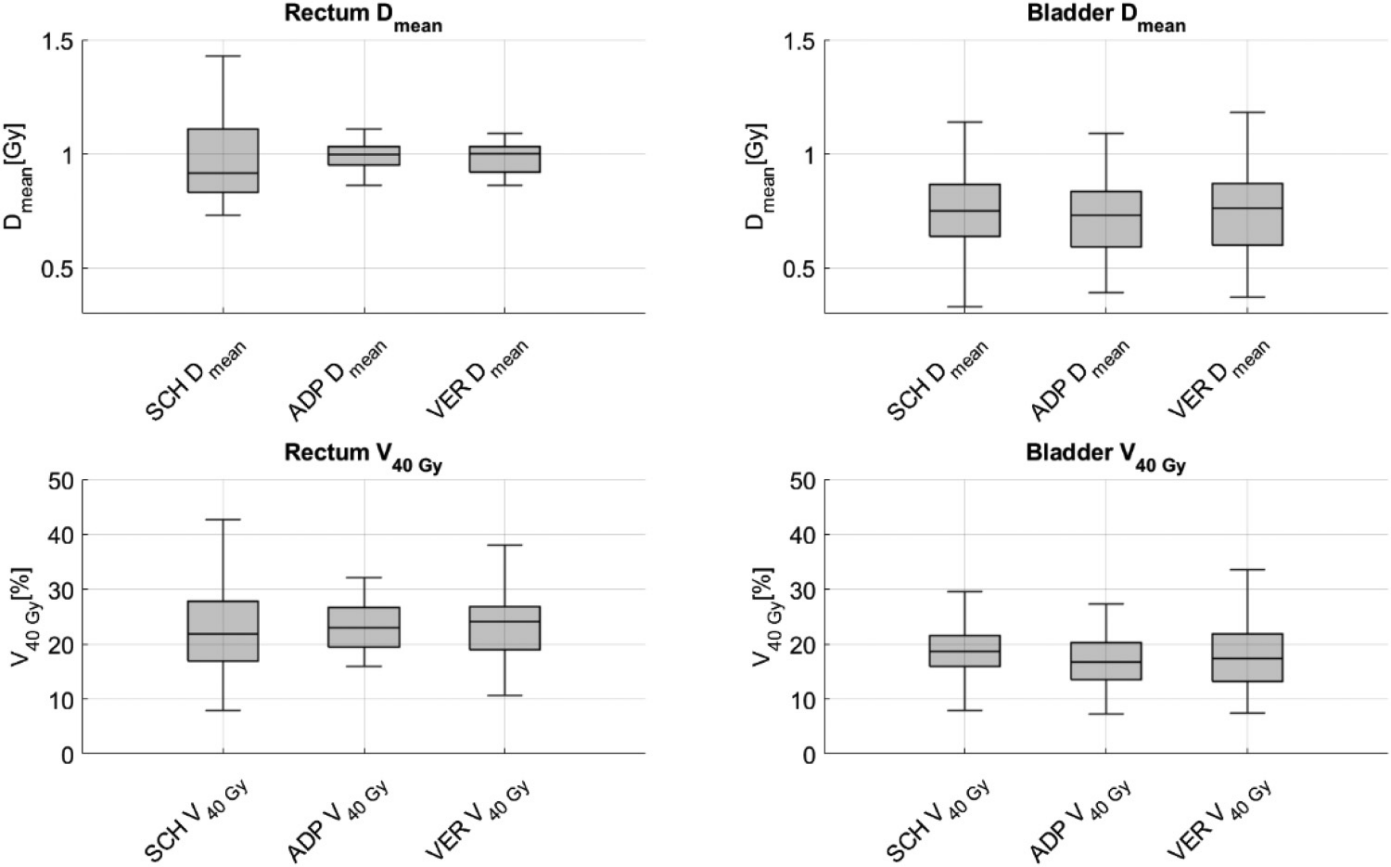
Dose exposure to the rectum and bladder, measured as Dmean and V_40Gy_, for scheduled (SCH), adapted (ADP), and verification (VER) plans.

The dose exposure to the bladder and rectum in terms of D_mean_ and V_40Gy_ is shown in Figure 8. When analyzing the parameters D_mean_ and V_40Gy_, statistically significant differences were observed for the bladder. For D_mean_, significant differences were found between SCH and ADP, as well as ADP and VER (p < 0.01 for both), and between SCH and VER (p = 0.02). The median bladder D_mean_ was 0.74 Gy/fx for SCH, 0.75 Gy/fx for ADP, and 0.76 Gy/fx for VER. For V_40Gy_, significant differences were observed between SCH and ADP, as well as ADP and VER (p < 0.01 for both). The median V_40Gy_ was 18.6% for SCH, 16.75% for ADP, and 17.65% for VER.

In terms of rectal exposure, no significant reduction was observed for the parameters D_mean_ and V_40Gy_. The median rectal D_mean_ was 0.91 Gy/fx for SCH, 0.99 Gy/fx for ADP, and 1 Gy/fx for VER. For the rectal V_40Gy_ the median was 21.75% for SCH, 23% for ADP and 24.1% for VER. When comparing the IQR between SCH and ADP as well as SCH and VER, it decreased for both D_mean_ and V_40Gy_. The IQR for rectal D_mean_ diminished from 0.26 for SCH to 0.08 for ADP and 0.11 for VER. Additionally the IQR for rectal V_40Gy_ dropped from 10.75 for SCH to 7.47 for ADP and 8.08 for VER.

The corresponding statistical data are presented in Table 5.

**Table 5.** The Table Presents Statistical Data for the Bladder and rectum (D_mean_ and V_40Gy_) for SCH, ADP, and VER Plans. Median, First Quartile (Q1), Third Quartile (Q3) Interquartile Range IQR (Q3-Q1) and p-Values (Wilcoxon Signed-Rank Test) are Included.

	Parameter	Plan	Median [Gy/fx] for D_mean_ [%] for V_40Gy_	Q1 [Gy/fx] for D_mean_[%] for V_40Gy_	Q3 [Gy/fx] for D_mean_ [%] for V_40Gy_	IQR	p-Value (to SCH)	p-Value (to ADP)
Rectum	D_mean_	SCH	0.91	0.84	1.10	0.26
		ADP	0.99	0.95	1.03	0.08	0.33
		VER	1.00	0.92	1.03	0.11	0.5	0.14
	V_40Gy_	SCH	21.75	16.90	27.65	10.75
		ADP	23.00	19.23	26.70	7.47	0.16
		VER	24.10	18.95	27.03	8.08	0.12	0.09
Bladder	D_mean_	SCH	0.74	0.63	0.90	0.27
		ADP	0.75	0.60	0.85	0.25	<0.01
		VER	0.76	0.59	0.88	0.29	0.02	<0.01
	V_40Gy_	SCH	18.60	15.90	22.03	6.13
		ADP	16.75	13.85	20.78	6.93	<0.01
		VER	17.65	13.50	22.30	8.80	0.08	<0.01

### Treatment Time

In total, 36 fractions from six patients undergoing conventional IGRT during the same time period were included and compared with 153 fractions treated using oART. Median treatment duration was 530 s (Q1: 486 s; Q3: 634 s) for IGRT and 1140 s (Q1: 960 s; Q3: 1260 s) for oART. This difference reached statistical significance (Mann–Whitney U test, two-sided, p < 0.01).

## Discussion

### Patient Selection

Patient enrollment in this study was primarily based on the availability of open slots at the linear accelerator, allowing for a flexible selection process while ensuring the efficient use of treatment resources.

### Target and Organs at Risk

oART resulted in improved target coverage and increased dose consistency across fractions, alongside enhanced sparing of bowel and bladder structures. Compared to scheduled plans, adaptive and verification plans achieved significantly higher CTV and PTV coverage and demonstrated reduced IQRs, indicating greater reproducibility. Adaptive workflows led to a consistent reduction in bowel D_0.1cc_ and bladder V_40Gy_ across SCH, ADP, and VER phases. While the bowel dose remained significantly lower throughout all phases, the bladder dose showed a significant reduction between SCH and ADP, which was not maintained in VER compared to SCH. Rectal dose remained largely unchanged, though variability decreased. Despite considerable individual variation, prostate volume showed no consistent trend across the cohort.

The oART led to measurable improvements in target coverage and sparing of OARs when compared to scheduled planning approaches. Dose coverage for CTV and PTV increased notably in ADP and VER plans relative to SCH, while remaining within clinically acceptable limits, even in the presence of minor decreases between ADP and VER. A reduction in the IQR between SCH and both ADP and VER was observed for the two volumes, indicating improved reproducibility of dose delivery across treatment fractions. These findings suggest that adaptive planning workflows allow for more robust realization of intended dose distributions, even under daily anatomical variation. Comparable results have been observed in other studies, highlighting the effectiveness of adaptive strategies in maintaining dosimetric accuracy despite anatomical changes over the course of treatment.^14^ The VER plans, calculated on the post-adaptation CBCT to account for intrafractional changes, demonstrated preservation of the dosimetric benefit. The enhanced precision of the adaptive workflows is further supported by the observed stability in D_2%_ values across all phases (SCH, ADP, VER). Although a slight increase in D_2%_ was noted from SCH to VER, dose levels remained consistently within acceptable thresholds. This demonstrates that adaptive planning effectively maintains target dose conformity without exceeding critical dose constraints, even with minor fluctuations, reinforcing the stability of the approach when applied under clinical conditions.

OAR sparing was most prominent for the bowel and bladder. D_0.1cc_ for the bowel differed significantly in all comparisons involving ADP and VER, with median values indicating substantial dose reduction. Although the IQR for VER was slightly increased compared to ADP, this effect may be attributed to anatomical shifts occurring between adaptation and beam-on. Nonetheless, the overall dose distribution supports improved bowel protection with oART, consistent with previous observations in pelvic and abdominal tumor sites.^15,16^ High-dose subvolume reduction, particularly in bowel structures, is considered relevant for late toxicity, making this improvement clinically meaningful.

For the bladder, statistically significant differences in D_mean_ were observed across all planning strategies, with a slight increase from scheduled to adaptive plans. This modest rise likely reflects prioritization of target coverage during daily re-optimization and is unlikely to be of clinical consequence. In terms of high-dose exposure (V_40Gy_), significant differences were found between scheduled and adaptive strategies, particularly between SCH and ADP as well as between ADP and VER, while no significant difference was observed between SCH and VER.

These findings suggest that bladder sparing is most effectively achieved through fully adapted workflows, likely due to improved accommodation of inter-fractional anatomical variation. This supports the clinical potential of oART in reducing high-dose burden to the bladder and enhancing treatment tolerability.^15^

The rectum demonstrated less variability between planning strategies. While D_mean_ and V_40Gy_ remained relatively consistent across the groups, a trend toward a reduced interquartile range (IQR) was observed in both ADP and VER plans compared to SCH. This suggests that adaptive planning strategies, despite maintaining similar average dose metrics, may enhance the consistency of dose distributions across treatment fractions by reducing variability. These findings are in line with observations from Shelley et al (2023) and Åström et al (2022), who demonstrated that CBCT-based oART improves anatomical alignment and dose precision through daily adaptation. Shelley et al focused on cervical cancer, while Åström et al investigated bladder cancer - both anatomically variable regions. Despite differences in tumor sites, the consistent finding of improved dose stability supports the broader applicability of oART in reducing variability in regions prone to inter-fractional changes, such as the rectum in prostate treatments.^16,17^

The analysis of target coverage also points to the continued relevance of PTV margins. Even SCH plans maintained CTV D_98%_ above 97%, suggesting that the conventional margin approach offers a baseline level of robustness against interfractional variation. However, adaptive planning consistently improved D_98%_ values and reduced variability, which may be especially beneficial for patients with outlier anatomy. VER plans further demonstrated that intrafractional changes did not negate the advantages gained through adaptation, although small degradations in coverage between ADP and VER highlight the importance of minimizing time between imaging and treatment.

These findings indicate that adaptive workflows could allow for reevaluation of current margin concepts. As daily anatomical adaptation compensates for interfractional variation, the conventional isotropic PTV margin may become less critical. Future approaches might therefore focus on individualized or anisotropic margins or even margin-free adaptive strategies to further improve treatment precision and organ sparing.

One of the strengths of the current analysis is the inclusion of all three plan types SCH, ADP, and VER per patient, enabling detailed intra-individual comparisons. The observed improvements in reproducibility, as captured by IQR metrics, extend beyond mean dose values and provide insight into the overall stability of the delivered dose across fractions. By integrating VER plans, the analysis captures the real-world performance of adaptive plans under conditions that include intrafractional variation. The findings also demonstrate that the adaptive workflow is applicable even under clinical constraints, with patient inclusion determined pragmatically by machine availability.

Limitations include the non-randomized patient selection based on available treatment capacity, which may introduce selection bias. The sample size, while sufficient to demonstrate major dosimetric trends, does not allow for conclusions regarding rare anatomical configurations or extreme motion patterns. Moreover, as this is a single-center investigation, external validity and generalizability remain limited and warrant confirmation in larger multicenter studies. Furthermore, the analysis focused exclusively on dosimetric parameters without correlating to clinical endpoints such as toxicity or tumor control. While improved dose distributions represent a prerequisite for clinical benefit, prospective studies are needed to validate these assumptions. An example of such an approach is currently being pursued in the PRoART study (Pelvic cancer Registry for online Adaptive Radiotherapy; ClinicalTrials.gov, NCT06185062), which is being conducted at several German centers and aims to link adaptive dosimetric improvements with patient-reported and clinical outcome data.

Intrafractional motion management was not part of the workflow, and while CTV and PTV coverage could be evaluated in the VER plans, these plans only partially reflect deviations occurring after the initial CBCT, as they provide a single post-adaptation snapshot without capturing the full temporal extent of intrafractional motion. Real-time tracking or motion monitoring could potentially improve plan robustness further, especially in patients with significant organ motion during treatment. In addition, while plan adaptation occurred within a clinically acceptable time frame, treatment duration was not formally analyzed. Temporal delays between imaging and treatment initiation in adaptive radiotherapy workflows have the potential to impact dose distribution accuracy, highlighting the importance of further exploration in this context.

### Organ Volumes

Prostate volume exhibited substantial inter-individual variability throughout the course of conventionally fractionated radiotherapy, without demonstrating a consistent temporal trend at the cohort level. Median volumetric measurements remained stable over the treatment period, with individual increases and decreases effectively balancing each other. This stability suggests that population-based averages may obscure patient-specific fluctuations that do not accumulate to a net volumetric change.

In contrast to our findings, studies investigating hypofractionated radiotherapy have reported significant prostate volume increases during treatment, indicating that interfractional swelling is a common phenomenon in these regimens.^18,19^ This observation highlights the potential for dose discrepancies when interfractional changes are not adequately accounted for. Similarly, CBCT-based analyses during conventionally fractionated radiotherapy have shown daily volumetric variations of the prostate, rectum, and bladder, which can lead to deviations from planned dose distributions if not monitored closely.^20^

Although no systematic volumetric increase was observed in our cohort, the observed inter-fraction variability underlines the importance of precise anatomical monitoring throughout the treatment course. The implementation of oART represents a promising strategy to account for patient-specific anatomical deviations, even in the absence of significant volumetric changes. By enabling real-time adjustments to the treatment plan, oART optimizes target coverage and minimizes exposure to surrounding organs at risk. This adaptive approach could be particularly beneficial in conventionally fractionated protocols, where fixed population-based margins may not sufficiently account for daily anatomical shifts.

### Time

An additional dimension of this study is the temporal aspect of treatment delivery. As expected, the adaptive workflow required more time than conventional IGRT, with median durations of approximately 19 min [1140 s] for oART compared to less than 9 min [530 s] for IGRT. While this represents a substantial increase, the extended session length must be viewed in the context of the dosimetric improvements achieved by daily plan adaptation. Similar findings have been reported in previous studies for bladder cancer treatments, reinforcing the notion that oART entails an additional time investment but provides a measurable gain in treatment precision and reproducibility.^21^ Importantly, the prolonged treatment time did not preclude clinical feasibility, as all fractions were completed successfully in routine practice. Moreover, continued advances in automation, artificial intelligence, and workflow integration are likely to further streamline adaptive sessions, potentially narrowing the gap to IGRT in the near future. Thus, although current oART workflows are more time-consuming, the additional duration can be justified by the improvements in target coverage and treatment consistency, supporting its integration into everyday clinical care.

## Conclusion

This study demonstrates that CBCT-based oART improves target dose coverage for prostate cancer while ensuring treatment precision and stability. The ability to dynamically adjust the dose distribution in response to anatomical changes highlights oART's potential to optimize prostate radiotherapy, particularly in the presence of inter- and intrafractional variations. Beyond improving target coverage, adaptive workflows contribute to enhanced protection of surrounding organs, particularly the bladder and bowel, by allowing for more precise dose modulation.

These findings reinforce the clinical relevance of oART for prostate cancer and support its continued development as a patient-specific, precision-enhancing radiotherapy technique.
